# Mr. Harrold's Case of Fractured Vertebræ

**Published:** 1811-03

**Authors:** C. Harrold

**Affiliations:** Cheshunt, Herts


### *To the Editors of the Medical and Physical Journal.*

### *Mr. Harrold's Case of Fractured Vertebræ.*

Gentlemen,

The fifteenth volume of the Medical and Physical Journal contains two Letters of mine on the subject of fractures of the thigh, with a pretty full description and accompanying plate of a machine, invented by me, for the more successful management of such cases, and a short history of a case of fracture of both thighs, where the use of this instrument was followed by very satisfactory success.

As I had bestowed much time, labour and thought upon this contrivance, which I flattered myself would prove useful to the world, I readily confess that I have been disappointed in not having seen so important a subject as the best mode of treating fractures of this description discussed in your Journal*.

The present communication, however, more immediately relates to the application of this machine to another purpose, that of *Fractures of the Spine*.

In page 10 of the 15th volume of your Journal, I observed, that it would be worth the trial in these melancholy cases; and I have lately had an opportunity of so employing it.

There may be much diversity of opinion concerning the value of life prolonged, when subjected to some serious privations and inconveniencies; but there are, doubtless, many

---

* We feel indebted to Mr. Harrold for thus recalling the attention of the faculty to his ingenious invention for the security of the fractured *os femoris*. Though we fully admit the principle upon which Mr. Pott founded his method, yet we must acknowledge the difficulties which accompany the application of it to practice, and confess that we have too frequently seen deformity result from placing the fractured limb on the side. Ed.

situations where life, if not valuable to the individual on his own account, becomes so in relation to his friends, or to the world at large; especially where the intellectual powers are unimpaired, and where there is not much bodily suffering. Besides, in the imperfect and acknowledged slow progression of medical science, if it have no other value, it may have that of being one step in advance towards a more happy issue.

CASE.

On the 8th of June, 1810, I was called, at the distance of four miles, to Henry Newland, an athletic labouring man, 28 years of age, who, before his present accident, had, and appeared to have good health, with the exception of some scars about the throat, denoting scrophulous affection which had existed in his childhood. He had been at work in an open chalk-pit, and the vault, about fifteen feet high, suddenly giving way, had buried him several feet under chalk and large flints. He was speedily dug out and conveyed in a cart (the wheels of which, being new had large prominent nails in the rim) about two miles over a hard road, to the place where I saw him. He was in bed, and there was, very evidently, a fracture of the last dorsal and first lumbar vertebræ; for the back there formed a considerable angle, and there was a complete paraplegia.

I had seen but one case of the kind before, which terminated fatally in about six weeks; and the death of that patient appeared to me to be occasioned, not so much by the mere fracture of the vertebræ, and consequent pressure and injury of the medulla spinalis, as by there being no opportunity afforded (from the frequent and necessary motion of the patient for purposes of cleanliness, &c.) for the fractured portions of bone to unite properly, if at all, and from the gangrene and destruction of parts which, from their want of feeling, gave no warning of their danger.

The first night my patient lay upon a bed, but he suffered great pain in his back, had no rest, and had made use of the head curtains to assist him in varying his position, in the fruitless hope of obtaining ease.

The next morning he was carefully moved into the fracture bed, his legs and thighs supported by the fracture boxes, a little bent at the knees; and the sacrum resting upon the moveable cushion over the trap-door of the machine, designed for the convenient use of the bed-pan.

It became necessary to pass the catheter every day, as he had no discharge of urine without it, and, as in this position of the patient, a good deal of the water unavoidably ran down down by the side of the instrument, and would have wetted his moveable mattress, the latter was constantly, during the operation, removed, and the bed-pan substituted, and the discharge of water assisted by pressure upon the region of the bladder.* He never felt the passage of the catheter into his bladder, and seldom much uneasiness from distension till a short time before the usual hour of passing it, except when he had been tempted by some of his acquaintance (and unfortunately, in this instance, he was at a public house) to exceed his usual allowance of liquids, when I have sometimes drawn off two pints and a half of urine. Mischief, however, was going on in the bladder, for after some time there was a good deal of offensive purulent matter discharged, after the thinner part of the urine was removed; and I had reason to regret (as it was impossible for me, at that distance, to see him more than once a day) that I had not used a flexible catheter, and left it in the bladder. But, as in the course of a few weeks, this discharge ceased, and he can now retain a pint or a pint and half of urine at a time, and discharge it when he pleases, some good may have resulted from the evil (though it is an experiment I should not willingly repeat), for Mr. Hey observes, "I have tried these different methods so often, and in cases so nearly similar, that I can scarcely entertain a doubt, that a person regains the power of expelling his urine much sooner when the catheter is withdrawn after each operation, than when it is left in the urethra." P. 399, 1st edit.

It was some time before my patient knew the exact position of his legs; for, though they were perfectly insensible to pinching, &c. he still had a sort of consciousness of their existence (*somewhat* similar perhaps to that which a person has of an amputated limb, from the nerves proper to it still existing), for they sometimes felt as if they were *floating* about, and at other times as if they were laid across each other, and he had sometimes a good deal of pain extending down the right thigh, which was apparently relieved by friction. Except what I have mentioned, he suffered no pain upon the machine during the whole time that he was upon it, unless when his bowels were once or twice disordered during his confinement.

---

* Much trouble and some mischief might be prevented by a tube made to fit into the catheter (after its introduction) at a right angle, which might pass between the thighs, and by acting as a syphon, not only prevent the escape of the urine by the side of the instrument, but render pressure upon the bladder, and, consequently, upon the lower part of the spine and sacrum unnecessary.

He had nothing under him but the hair mattress, upon which his spine rested perfectly straight; his head and shoulders elevated about three inches, and, to ease the pressure in a small degree upon the fractured part, a worsted stocking once doubled was gently pressed a little way under, opposite the fracture on each side.

Notwithstanding all our care, the death of the integuments over the sacrum could not be prevented; for, it must be remembered, that there was no feeling of injury, and no power, by any slight motion, to lessen, what might be called, a dead weight.* To obviate this as much as possible, and at the same time to dress the wound more effectually, at the expiration of the month he was moved into a bed by six men, one to each limb and one on each side, steadily supporting the rolled ends of a broad towel which had been previously passed under the back. We were then enabled to turn him every day (at first very carefully, to prevent any twist of the spine) to dress his wound, which went on favourably (though its progress was long interrupted by pressure), and was perfectly healed by the latter end of October: during the last two months he generally dressed it himself.

His present state is this—his back is as straight and flexible, and apparently as strong as ever; for he can sit perfectly erect in a chair, and can stoop sufficiently, as he sits, to rub his feet upon the ground; and the only mark of fracture remaining is that of the spinous processes of the fractured vertebræ being a very little more prominent than the rest. He retains and passes his urine as he pleases, but I suspect that the abdominal muscles are much more concerned in expelling its contents, than the muscular coat of the bladder. He commonly has a stool once in three or four days, and then always at the time he is expelling the contents of the bladder; and except when his stools are liquid, he *now* begins to be conscious of their passage.

He has hitherto recovered no feeling or power, in themselves, of moving his legs and thighs, for even the glutei muscles do not perform their office; but his appetite and

* Pressure seems so apt, in these cases, soon to produce destruction of the integuments of the nates, that, but for the speedy reproduction of the destroyed parts, I should be disposed to consider it as arising from diminished vital energy; for, in cases of fracture of the thigh upon the same machine, I have not seen the smallest tendency even to inflammation; perhaps, for *this* purpose a cushion of wool might be better than horse-hair; but, whatever the material, it must possess sufficient firmness to preserve a proper position.

bodily bodily health and spirits are good, and he is so active as to have made a practice, not only of dressing himself entirely, but for some time of letting himself, step by step, down stairs; by which means he has produced a wound over the tuberosity of the left ischium: this, however, is now healing, and he is about to receive instruction in the business of a taylor.

How much more, in the course of time, *nature* may do for him, it may perhaps, be difficult to conjecture; but as the case appears to me very interesting in various views, I shall be happy to see any new light thrown upon the subject by some of your ingenious and experienced correspondents.

C. HARROLD.

*Cheshunt, Herts, Jan.* 10, 1811.

*P.* S. I should have observed, in its proper place, that soon after my patient was removed from the machine into a bed, there was some œdema of the legs and thighs, and that though friction of them was not felt, it was followed by a sense of warmth and by perspiration of the parts; and that, after a while, a compleat desquamation of the cuticle of the legs, thighs and feet followed.

I have also observed, when upon the machine, that, though there was no feeling in the penis, there was sometimes evident action of the cremaster muscle.

